# Levothyroxine for the Treatment of Subclinical Hypothyroidism and Cardiovascular Disease

**DOI:** 10.3389/fendo.2020.591588

**Published:** 2020-10-21

**Authors:** Laura Y. Sue, Angela M. Leung

**Affiliations:** ^1^Division of Endocrinology, Diabetes, and Metabolism, Department of Medicine, UCLA David Geffen School of Medicine, Los Angeles, CA, United States; ^2^Division of Endocrinology, Diabetes, and Metabolism, Department of Medicine, VA Greater Los Angeles Healthcare System, Los Angeles, CA, United States

**Keywords:** subclinical hypothyroidism, cardiovascular disease, levothyroxine, thyroid disease, thyroid treatment

## Abstract

Subclinical hypothyroidism is a biochemical condition defined by elevated serum thyroid-stimulating hormone levels in the setting of normal levels of the peripheral thyroid hormones, thyroxine and triiodothyronine. Thyroid hormones act on the heart through various mechanisms and subclinical hypothyroidism has been associated with risk factors for cardiovascular disease, such as hypertension and dyslipidemia. In addition, evidence from multiple studies supports an association between subclinical hypothyroidism and cardiovascular disease. However, the use of levothyroxine in subclinical hypothyroidism to reduce cardiovascular disease risk is not clearly beneficial. Treatment with levothyroxine may only provide benefit in certain subgroups, such as patients who are younger or at higher risk of cardiovascular disease. At present, most of the international societal guidelines advise that treatment decisions should be individualized based on patient age, degree of serum thyroid-stimulating hormone (TSH) elevation, symptoms, cardiovascular disease (CVD) risk, and other co-morbidities. Further study in this area is recommended.

## Introduction

Subclinical hypothyroidism (SCH) is a biochemical condition defined by elevated serum thyroid-stimulating hormone (TSH or thyrotropin) levels in the setting of normal levels of the peripheral thyroid hormones, thyroxine and triiodothyronine (1). Mild grade 1 SCH (upper limit of TSH 9.9 mIU/L) can be differentiated from more severe grade 2 SCH (TSH ≥10 mIU/L), with approximately 80–90% of SCH patients falling in the grade 1 category (1–3). Most commonly, SCH is caused by autoimmune thyroiditis but can be due also to other causes. Laboratory data in the recovery phase of thyroiditis, in the course of medication, and in the elderly are similar to SCH (1, 2) and may complicate the decision to treat with levothyroxine.

Symptoms of SCH may include fatigue, cold intolerance, weight gain, and constipation, as well as reduced mood, quality of life, cognitive function, and memory (1). The clinical symptoms are usually milder in individuals with SCH than those with overt hypothyroidism. They can be absent in individuals with grade 1 SCH and are expected to increase in frequency and severity with increasing serum TSH concentrations (1, 2). Although it may vary by age, sex, race/ethnicity, and geography, the reported prevalence of SCH ranges from 0.4–16.9% in one review (4) to as high as 19.7% (5) and 20% (6) in other series. The prevalence of SCH increases with age and is highest among women, elderly, and those living in iodine-deficient regions (1–3, 7, 8).

The risk of progression from SCH to overt hypothyroidism is 3.3–11.4% per year (9, 10). Higher rates of progression are seen in those with grade 2 SCH (compared to grade 1 SCH), positive serum thyroid peroxidase (TPO) antibody titers, female sex, high baseline serum TSH levels, and baseline free T4 (FT4) levels at the lower end of the reference interval (1, 2, 9–13). In a Japanese population of elderly atomic bomb survivors, those with SCH had more than 4.5 times increased risk of progression to overt hypothyroidism, compared with controls, with a baseline TSH greater than 8 mIU/L predictive of progression; however, TSH levels also normalized in 53.5% of patients over the study period (14).

## Pathophysiology of Cardiovascular Disease (CVD) Related to SCH

Thyroid hormones act on the heart through various mechanisms. On a cellular level, thyroid hormone regulates cardiac gene expression through its actions on cardiomyocytes and in the activity of ion channels (sodium, calcium, potassium) in the cardiomyocyte cell membrane; thyroid hormones also influence the cardiovascular system through their effects on peripheral circulation (15, 16). Thyroid hormone receptors can be found in the myocardium and in vascular endothelium, which allows for regulation of these tissue processes, including endothelial nitric oxide production and vascular tone (16).

SCH has been associated with various risk factors for CVD, such as hypertension and dyslipidemia. Increased blood pressure in SCH may be a result of reduced thyroid hormone-mediated endothelial-dependent vasodilation (16). Specific to dyslipidemia, decreased activity of both lipoprotein lipase activity in adipose tissue and hepatic lipase activity in the liver are thought to contribute to increased serum triglyceride levels (17). A reduced number of low-density lipoprotein (LDL) receptors and decreased cholesterol breakdown may also explain the dyslipidemia seen in SCH (16). In addition to increasing metabolic risk factors, SCH may negatively impact cardiac function itself, with studies demonstrating a possible role of liothyronine (T3) in mitochondrial function and repair/damage (18) and the improvement of cardiac output and reduction of peripheral vascular resistance upon T3 administration (19). Other studies have found that SCH is also associated with decreased ejection fraction, decreased arterial compliance, and increased risk of heart failure, possibly through increased renin-angiotensin-aldosterone axis activation, increased vasoconstriction, increased sympathetic activity, and reduced renal blood flow and glomerular filtration rates (16, 17).

## Associations Between SCH and CVD

Numerous studies have explored the possible associations between SCH and CVD, a leading cause of death worldwide that accounts for one third of all deaths (20). The major studies on this topic will be reviewed here. Of note, the relationships between SCH, serum TSH levels, and specific risk factors for CVD, such as blood pressure (6, 21, 22), glucose levels (6), and cholesterol levels (6, 23–27), have also been examined but will not be reviewed in detail.

Evidence from multiple studies supports an association between SCH and CVD. Early studies suggested an association between SCH and CVD, but sample sizes were small (28, 29). In an observational study of postmenopausal women in the Netherlands, those with SCH had an increased prevalence of myocardial infarction [odds ratio (OR) 2.3, 95% confidence interval (CI) 1.3–4.0]; this risk was even higher among those with positive serum TPO antibodies (OR 3.5, 95% CI 1.7–7.4) (30). Women with SCH, either with or without the presence of TPO antibodies, also had a higher risk of aortic atherosclerosis (30). The calculated population attributable risk of SCH to myocardial infarction in the study was 14%, similar to those calculated for hypertension, diabetes, smoking, and hypercholesterolemia (14–18%) (30). In another study of Medicare patients (age 65 years or older) with SCH, those with a TSH greater than 10 mIU/L had a significantly higher prevalence of coronary heart disease compared with those whose TSH was less than or equal to 4.6 (52.6% versus 25.0%; p = 0.007) (31). In a Danish primary care population of 1,212 patients age 20–69 years without previous thyroid disease, biochemical SCH was associated with increased CVD, but only among men younger than 50 years old (OR 3.3, 95% CI 1.6–6.8) (5).

An Australian study of 2,108 patients (mean age, 50 years), was the first longitudinal study to demonstrate a positive relationship between SCH and cardiovascular events [hazard ratio (HR) 1.7, 95% CI 1.2–2.4, p <0.01], which persisted after adjustment for cardiovascular risk factors and pre-existing thyroid disease or goiter (HR 1.8, 95% CI 1.7–2.7, p <0.01), suggesting that the association may be driven by mechanisms other than established cardiovascular risk factors (32). Other cohort studies have shown similar associations, such as a study among adults in Taiwan without baseline thyroid disease [relative risk (RR) of cardiovascular death 1.68, 95% CI 1.02–2.76, p <0.05] (33) and a re-analysis of the Whickham Survey cohort (HR of ischemic heart disease 1.76, 95% CI 1.15–2.71, p = 0.01) (21), although the original analysis had shown no association between SCH and CVD (9) and the association was not significant when levothyroxine treatment was removed from the model (21).

HUNT, a large prospective population-based cohort study of 25,313 individuals in Norway, found that TSH levels within the reference range were significantly positively associated with risk of incident cardiovascular death in the total population (P for trend 0.01) and among women (HR 1.41, 95% CI 1.02–1.96 for TSH 1.5–2.4 mIU/L; HR 1.69, 95% CI 1.14–2.52 for TSH 2.5–3.5 mIU/L; compared with TSH 0.50–1.4 mIU/L; P for trend 0.005) over a median follow-up of 8.3 years (34). The positive association among women was attenuated by adjustment for blood pressure, serum cholesterol levels, body mass index, serum creatinine level, and use of antihypertensive medications, but remained statistically significant (HR 1.30, 95% CI 1.06–1.60). There was no association between TSH values above the reference range (in participants with SCH) and cardiovascular death. An update performed at 12 years of follow-up among the HUNT study participants again demonstrated an association between baseline TSH in the higher end of the reference range and incident cardiovascular mortality among women (HR 1.41, 95% CI 1.06–1.87 for TSH 1.5–2.4 mIU/L; HR 1.45, 95% CI 1.01–2.08 for TSH 2.5–3.5 mIU/L; compared with TSH 0.50–1.4 mIU/L; P for trend 0.005) and between SCH and cardiovascular mortality among women (HR 1.76, 95% CI 1.21–2.56); however, this increased risk did not translate into higher risk of hospitalization for myocardial infarction (35).

Among those with higher baseline CVD risks, the presence of SCH may further increase the risk of cardiovascular outcomes. Cardiac patients with SCH who were admitted to an Italian hospital had 2.4 times higher risk of cardiac death (95% CI 1.36–4.21, p = 0.02), compared with patients who were euthyroid (36). In Korean patients with high cardiovascular risk, defined by an Atherosclerotic Cardiovascular Disease (ASCVD) risk score >7.5% or known CVD, and TSH values in the highest quartile (>6.57), the risk of cardiovascular events was 2.42 times higher (95% CI 1.35–4.33), compared with euthyroid patients (37). Data from a study of patients at high risk for ASCVD at the Cleveland Clinic Preventive Cardiology Clinic revealed higher all-cause mortality among untreated patients, those younger than age 65 years, and in those with serum TSH levels between 6.1–10 mIU/L or greater than 10 mIU/L (38).

In a large individual-participant pooled analysis of prospective cohort studies (n = 55,287) by the Thyroid Studies Collaboration, SCH and coronary heart disease were positively associated in adults with TSH 10–19.9 mIU/L (HR 1.89, 95% CI 1.28–2.80), compared with those with normal TSH levels, with the risk further increasing the higher the serum TSH level was (P for trend <0.001) (39). Another large pooled analysis of over 31,900 participants, also done by the Thyroid Studies Collaboration, found that the increased risk of coronary heart disease events in those with SCH did not vary by serum TPO antibody status (P for interaction = 0.65) (40). SCH has also been associated with all-cause mortality, mediated by CVD, in a study that used data from the National Health and Nutrition Examination Survey in the United States (41).

In contrast, multiple studies have found insufficient evidence to support an association between SCH and CVD. The Cardiovascular Health Study was a large prospective cohort study that enrolled 3,233 elderly patients, including 496 (15%) with SCH (42). There was no difference in prevalent coronary heart disease cases at baseline between patients with SCH (19.8%) and those who were euthyroid (18.5%). There was also no difference in incident coronary heart disease cases (HR 1.07, 95% CI 0.90–1.28), compared with euthyroid patients, or in death from cardiovascular causes (HR 1.16, 95% CI 0.92–1.46). In a follow-up study with more participants, including 4,184 euthyroid and 679 with SCH, there was again no association between persistent SCH and incident coronary heart disease (HR 1.12, 95% CI 0.93–1.36) or cardiovascular death (HR 1.07, 95% CI 0.87–1.31) (43).

In a subset of the MrOS study (n = 1,587), a prospective study in the U.S. of elderly men to evaluate healthy aging and fracture risk, having SCH increased the risk of cardiovascular death, but the number of participants with SCH was small and the associations were not significant [TSH <10 mIU/L: relative hazard (RH) 1.28, 95% CI 0.77–2.14; TSH ≥10 mIU/L: RH 3.32, 95% CI 0.82–13.45] (44). In a nested case-cohort study of the Women’s Health Initiative, there was no association between risk of myocardial infarction and SCH among postmenopausal U.S. women with varying degrees of SCH and serum TPO antibodies. Compared with euthyroid individuals, the HR for incident myocardial infarction was 0.90 (95% CI 0.47–1.74) among women with TSH of ≥7 mIU/L and positive serum TPO antibodies (45). SCH and cardiovascular events were also not associated in the Tehran Thyroid Study of 3,975 participants (mean age, 46.5 years; 189 participants with SCH) (HR 0.71, 95% CI 0.37–1.33) (46).

Several meta-analyses have summarized available data about SCH and the risk of CVD. Singh et al. examined several large cross-sectional and cohort studies published between 2000 through March 2006 (47). The meta-analysis found increased risk of prevalent coronary heart disease at baseline (RR 1.53, 95% CI 1.31–1.79; P <0.001; 5 studies) and incident coronary heart disease at follow up (RR 1.19, 95% CI 1.02–1.38; P <0.05; 3 studies) for those with SCH versus those without SCH. In addition, SCH was associated with higher risk of death from cardiovascular causes (RR 1.28, 95% CI 1.02–1.60; p <0.05; 3 studies) but not with all-cause mortality (RR 1.12, 95% CI 0.99–1.26; 3 studies) at follow-up. In a larger meta-analysis of published studies from 1977–2007 by Razvi et al. (48), the prevalence of ischemic heart disease was 23% higher in the SCH group compared with euthyroid subjects (95% CI 1.02–1.48; p = 0.03; 12 studies). Due to significant heterogeneity, however, subgroup analyses were performed; these analyses showed that the elevated risk of ischemic heart disease was only present in the subgroup of individuals younger than 65 years old (OR 1.57, 95% CI 1.19–2.06; p = 0.001; 7 studies) and not in the subgroup of individuals 65 years or older (OR 1.01, 95% CI 0.87–1.18; p = 0.01; 5 studies). Similarly, using data from available longitudinal cohort studies, SCH was associated with higher incidence of ischemic heart disease (OR 1.68, 95% 1.27–2.23; p <0.001; 3 studies) and cardiovascular mortality (OR 1.37, 95% CI 1.04–1.79; p = 0.02; 3 studies) but only among individuals younger than 65 years old.

A separate meta-analysis by Ochs et al. (49) suggested a mild positive association between SCH and coronary heart disease (RR 1.20, 95% CI 0.97–1.49; 10 studies), but this was again only statistically significant among younger individuals. Those with SCH who were younger than 65 years old had a 51% increase in risk of coronary heart disease (95% CI 1.09–2.09; 4 studies), compared with euthyroid individuals, versus an insignificantly increased risk for those with SCH who were 65 years or older (RR 1.05, 95% CI 0.90–1.20; 6 studies). Similar estimates were seen in subgroup analyses of those under versus those over age 60 years old. There was also a suggested association between SCH and cardiovascular mortality (RR 1.18, 95% CI 0.98–1.42; 8 studies) in this meta-analysis.

The most recent and largest meta-analysis to date by Moon et al. included 555,530 participants from 35 studies, with 11 studies including individuals at high cardiovascular risk (as defined by CVD risk factors or any disease that could increase risk of CVD) (50). Compared with euthyroid participants, those with SCH had a 33% higher risk of CVD (95% CI 1.14–1.54). As expected, this risk was even higher among those at high CVD risk (RR 2.20, 95% CI 1.28–3.77). Because previous studies had suggested a difference in CVD risk by age (51), the authors performed additional subgroup analyses: the RR of SCH-associated CVD was 1.54 (95% CI 1.21–1.96) among those with a mean age less than 65 years and 1.07 (95% CI 0.97–1.18) among those with a mean age of 65 years or older. Similar findings were seen in the Moon et al. meta-analysis for all-cause mortality, with higher risk in those with high CVD risk and additional analyses observing an association only among the younger subgroup.

## Use of Levothyroxine in SCH to Reduce CVD Risks

There are limited data regarding the use of levothyroxine for improving CVD outcomes in individuals with SCH. These studies are summarized in **Table 1**. The Whickham Survey in Great Britain, which had found increased risk of ischemic heart disease in those with SCH, was the first study to suggest that treatment with levothyroxine may be beneficial in SCH. While there was no difference in ischemic heart disease events or ischemic heart disease-related mortality, comparing those treated (n = 20) versus not treated (n = 71) among study participants with SCH, there was a significant difference in all-cause mortality, with an 78% lower rate of death among those treated with levothyroxine (p = 0.02) (21). In another much larger retrospective cohort analysis using data from the United Kingdom General Practitioner Research Database (52), treatment of SCH with levothyroxine was associated with a significant reduction in fatal and nonfatal ischemic heart disease events, death due to circulatory diseases (ischemic heart disease, cerebrovascular disease, peripheral vascular disease), and all-cause mortality. However, these associations were seen only in younger subjects (age 40–70 years, n = 3,093) and not in older subjects (age greater than 70 years old, n = 1,642). The multivariate adjusted HRs for ischemic heart disease events, death due to circulatory diseases, and all-cause mortality were 0.61 (95% CI 0.39–0.95), 0.54 (95% CI 0.37–0.92), and 0.36 (95% CI 0.19–0.66), respectively. The significant association between treatment with levothyroxine and decreased ischemic heart disease events was again seen only in younger individuals when additional analyses were performed: 1) in a restricted dataset of only those with persistent SCH during the follow up-period in the untreated group, after exclusion of those who started levothyroxine treatment after SCH had already progressed to overt hypothyroidism; and 2) stratification of participants by serum TSH <6.6 mIU/L and TSH ≥6.6 mIU/L. In sensitivity analyses stratifying the study population by age per decade, the greatest and only statistically significant reduction in fatal and nonfatal ischemic heart disease events was seen in the 61-70 year old age group (HR 0.41, 95% CI 0.17–0.97). To date, there has been no large, randomized, placebo-controlled trial demonstrating a benefit of treatment with levothyroxine in SCH on CVD risk.

**Table 1:** Summary table of the use of levothyroxine in SCH to reduce CVD risks.

Authors	Year of Publication	Sample Size	Study Design	Population	Results
Razvi et al. (21)	2010	91	Cohort study	Middle-aged community-dwelling adults with SCH enrolled in the Whickham Study, a population-based cross-sectional study in northern England with 20 years of follow up.	Reduced all-cause mortality in those treated with levothyroxine: HR 0.22, 95% CI 0.06–0.81 (p = 0.02).
Razvi et al. (52)	2012	4,735	Cohort study	3,093 individuals age 40–70 years and 1,642 individuals age 71–107 years old with SCH from the United Kingdom General Practitioner Research Database. The median levothyroxine dose was 75 mcg daily.	In younger individuals (age 40–70 years) who received treatment with levothyroxine, there were fewer ischemic heart disease events (HR 0.61, 95% CI 0.39–0.95), deaths due to circulatory diseases (HR 0.54, 95% CI 0.37–0.92), and deaths due to any cause (HR 0.36, 95% CI 0.19–0.66).
Andersen MN et al. (53)	2015	12,212	Cohort study	Primary care adult patients 18 years or older in Copenhagen, Denmark diagnosed with SCH between 2000 and 2009	No association between treatment with levothyroxine and myocardial infarction, CVD death, or all-cause mortality. Benefit of levothyroxine treatment for all-cause mortality in patients less than 65 years old (incidence rate ratio 0.63, 95% CI 0.40–0.99).
Anderson MN et al. (54)	2016	1,192	Cohort study	Adult clinic patients 18 years or older in Denmark with existing heart disease diagnosed with SCH from 1997 to 2011.	No association between treatment with levothyroxine and major adverse cardiac events, all-cause mortality, MACE, or hospital admissions.
Stott et al. (55)	2017	737	Randomized controlled trial	Adults 65 years or older from Scotland, Ireland, the Netherlands, and Switzerland with untreated SCH randomized to a median daily levothyroxine dose of 50 mcg or placebo.	No significant differences in fatal or nonfatal cardiovascular events (HR 0.89, 95% CI 0.47–1.69), new-onset atrial fibrillation (HR 0.80, 95% CI 0.35–1.80), or all-cause mortality (HR 1.91, 95% 0.65–5.60) between the levothyroxine and placebo groups.
Blum et al. (56)	2018	158	Randomized controlled trial	Subset of participants from the Stott et al. study: Adults 65 years or older from Scotland, Ireland, the Netherlands, and Switzerland with untreated SCH randomized to a median daily levothyroxine dose of 50 mcg or placebo.	No significant differences in mean carotid intima media thickness (p = 0.30), maximum carotid intima media thickness (p = 0.35), or maximum plaque thickness (p = 0.86) between the levothyroxine and placebo groups.

SCH, subclinical hypothyroidism; CVD, cardiovascular disease; HR, hazard ratio; CI, confidence interval; MACE, major adverse cardiovascular events.

In contrast, data from some studies suggest that levothyroxine plays no significant role in improving CVD outcomes in individuals with SCH. The Thyroid Hormone Replacement for Untreated Older Adults with Subclinical Hypothyroidism – A Randomized Placebo Controlled Trial (TRUST) included 737 adults aged 65 years old or older, who were randomized to a daily levothyroxine dose of 25 or 50 mcg (then titrated by serum TSH level; median daily dose 50 mcg) or placebo (55). There were no differences in the primary outcomes of hypothyroid symptoms or tiredness on a thyroid-specific quality of life questionnaire at 1 year. Although the study was not powered to detect a difference in CVD incidence or mortality, analysis of adverse events showed no significant differences in fatal or nonfatal cardiovascular events (HR 0.89, 95% CI 0.47–1.69), new-onset atrial fibrillation (HR 0.80, 95% CI 0.35–1.80), or all-cause mortality (HR 1.91, 95% 0.65–5.60) in those treated with levothyroxine, compared with those in the placebo group. Overall event rates were so low for death from CVD and heart failure that HRs were not able to be calculated. In a smaller randomized, double-blind, placebo-controlled trial nested within the TRUST study (56), 158 participants were randomized to levothyroxine (titrated to normalization of serum TSH) or placebo; there was no difference in carotid intima media thickness or carotid atherosclerosis, both predictors of CVD, with levothyroxine treatment. Taken together, these data suggest that treatment with levothyroxine may not improve CVD outcomes in SCH, but also does not seem to cause significant risk.

A large cohort analysis of a primary care population of Danish patients with SCH (n = 12,212) evaluated the effect of treatment with levothyroxine over a median follow up of 5 years (53). Treatment with levothyroxine was not associated with incidence of myocardial infarction, CVD death, or all-cause mortality in this population; sub-group analyses by younger/older individuals and grade 1/grade 2 SCH also did not demonstrate any significant differences with levothyroxine treatment, except in patients less than 65 years old with respect to all-cause mortality (incidence rate ratio 0.63, 95% CI 0.40–0.99). In a similar but smaller population of Danish patients with existing heart disease and SCH (n = 1,192), there was also no association between levothyroxine treatment and major adverse cardiac events, all-cause mortality, or hospital admissions (54).

Multiple studies have suggested improvement in cholesterol parameters, blood pressure, and various markers of cardiac and vascular structure/function with levothyroxine treatment in SCH (25, 57–61), but generally these studies were small, the results need to be replicated, and any improvement in CVD risk factors with levothyroxine treatment may not ultimately translate into a reduction in CVD risk. A recent double-blind, randomized controlled trial that enrolled patients with acute myocardial infarction and SCH in hospitals in the United Kingdom found no difference in the primary outcome of left ventricular ejection fraction at 52 weeks, comparing the 46 participants in the treatment group (levothyroxine 25 mcg daily titrated to serum TSH levels between 0.4–2.5) with the 49 participants in the placebo group (62). An older small study of 40 women with mild SCH randomized participants to 50 mcg of levothyroxine daily or placebo: after 6 months, there was no significant difference in cholesterol, triglycerides, LDL cholesterol, high-density lipoprotein (HDL) cholesterol, body mass index, or other parameters studied (63).

## Current Guidelines for the Treatment of SCH

A summary of guidelines on this topic from the various international organizations is provided in **Table 2**. The 2012 American Thyroid Association guidelines for hypothyroidism in adults (51) recommend starting thyroid hormone treatment for primary hypothyroidism when the serum TSH is above 10 mIU/L and considering treatment in those with increased CVD risk when the serum TSH is 4.5–10 mIU/L. While there are limited outcome data on treating patients with TSH 2.5–4.5 mIU/L, the guidelines refer to studies demonstrating improved markers of atherosclerosis risk (lipids, endothelial function, and intima media thickness) in the consideration for treatment of SCH with serum TSH values in this range. The guidelines note that in SCH, the initial levothyroxine dose is typically lower than that required in overt hypothyroidism and suggest a daily dose of 25–75 mcg, depending on the degree of TSH elevation and to be adjusted based on symptoms and serum thyroid function test monitoring. For those with known CVD, the 2014 American Thyroid Association guidelines for the treatment of hypothyroidism recommend initiating levothyroxine at a low dose, increasing slowly as needed, and observing closely for the development of cardiac symptoms (66). A working group organized by the National Heart, Lung, and Blood Institute in the United States in 2017 identified three areas for future research in thyroid-related CVD: 1) basic biology linking thyroid dysfunction to CVD and thyroid hormone action in cardiovascular tissues; 2) identification of specific thyroid patients who might benefit from preventive interventions or treatments for CVD; and 3) clinical trials using thyroid pathways or thyroid treatments to influence CVD outcomes (68).

**Table 2:** Summary of current guidelines for the treatment of SCH.

Society/Expert Panel	Year of Publication	Recommendations
American Thyroid Association (51)	2012	Primary hypothyroidism should be treated when the serum TSH is above 10 mIU/L and considered in those with increased CVD risk when the serum TSH is 4.5–10 mIU/L. The initial levothyroxine dose is typically lower in SCH than that required in overt hypothyroidism.
European Thyroid Association (64)	2013	Levothyroxine should be initiated in younger patients (age less than 65 years) who have serum TSH levels greater than 10 mIU/L and in younger patients with SCH who have symptoms consistent with hypothyroidism, even if the TSH is less than 10 mIU/L.
Latin American Thyroid Society (65)	2013	The decision to treat SCH should be based on the risk of progression to overt hypothyroidism. Levothyroxine should be initiated if the serum TSH is persistently greater than 10 mIU/L and considered if the serum TSH is 4.5–10 mIU/L in those younger than age 65 years with increased CVD risk.
American Thyroid Association (66)	2014	In those with SCH and known CVD, levothyroxine should be initiated at a low dose, increasing slowly as needed, and observed closely for the development of cardiac symptoms.
An international expert panel (findings published in the British Medical Journal) (67)	2019	Levothyroxine is generally recommended against for those with SCH, with the exception of women who are pregnant or trying to conceive or those with a serum TSH greater than 20 mIU/L. The recommendation may not apply to those with severe symptoms or those younger than 30 years old.

SCH, subclinical hypothyroidism; TSH, thyroid-stimulating hormone; CVD, cardiovascular disease.

The 2013 European Thyroid Association guidelines on the management of SCH (64) recommend levothyroxine treatment in younger patients (age less than 65 years) who have serum TSH levels greater than 10 mIU/L and in younger patients with SCH who have symptoms consistent with hypothyroidism, even when the serum TSH is less than 10 mIU/L. These guidelines advise that in older individuals, age-specific reference ranges should be used to diagnose SCH. Observation without treatment should be the strategy of choice in patients greater than 80–85 years old with SCH and serum TSH less than or equal to 10 mIU/L. Levothyroxine is the thyroid hormone formulation of choice and the dose should be weight-based in patients without cardiac disease and start at a low amount (25 or 50 mcg daily) in those with cardiac disease and/or older age.

The 2013 Latin American Thyroid Society hypothyroidism management guidelines (65) recommend starting treatment in SCH based on how likely an individual is to progress to overt hypothyroidism. Therefore, the authors recommend starting thyroid hormone in individuals with a serum TSH persistently greater than 10 mIU/L and considering treatment initiation in those with a serum TSH 4.5–10 mIU/L who are younger than age 65 years with increased CVD risk (previous CVD, diabetes, dyslipidemia, hypertension, or metabolic syndrome), especially if the TSH is persistently greater than 7 mIU/L. The guidelines note that treatment could be considered for those with persistent mild elevations in TSH if serum TPO antibodies are positive and ultrasound findings are consistent with autoimmune thyroiditis. A lower grade recommendation is made for a short trial of levothyroxine in symptomatic middle-aged patients and continuation of this therapy if a clear benefit is seen. The guidelines recommend against treatment of elderly (older than 65 years) or very elderly (older than 80 years) patients with SCH and TSH levels less than 10 mIU/L.

Lastly and somewhat controversially, Bekkering et al. published a British clinical practice guideline in 2019 (67), which suggested a lack of benefit from thyroid hormone treatment in nearly all those with SCH, and specifically that asymptomatic SCH patients or those with non-specific symptoms should not be treated. The recommendation did not apply to women who are pregnant or trying to conceive or those with a serum TSH greater than 20 mIU/L and may not apply to those with severe symptoms or those younger than 30 years old, as evidence is limited in these subgroups. Commentary of the guideline argues that not enough evidence was provided to support the recommendation not to treat and that the decision to initiate treatment should be individualized based on degree of serum TSH elevation, symptoms, patient preference, and other factors (69, 70).

## Discussion

The role of levothyroxine for reducing the risk of CVD in individuals with SCH remains unclear. While SCH has been associated with both CVD and CVD risk factors, this is not consistent across all studies and the risk of CVD may be only significant elevated in younger individuals. Data from small studies showing improvements in CVD risk factors and markers of CVD risk may suggest some benefit of levothyroxine treatment in SCH; however, it is unclear if this risk reduction would ultimately confer a CVD or CVD mortality benefit. Furthermore, in larger observational and randomized, placebo-controlled studies, there is not yet convincing, consistent evidence that treatment with levothyroxine leads to reductions in CVD outcomes.

At the current time, most of the international societal guidelines advise that treatment decisions should be individualized based on patient age, degree of serum TSH elevation, symptoms, CVD risk, and other co-morbidities. Caution must be taken in initiating levothyroxine treatment for SCH in the elderly. Of note, there are different reference intervals that are applicable for specific sub-populations (the elderly, pregnant women), which may affect the decision to treat or not treat with levothyroxine. Further study in this area should include larger studies powered to detect differences in CVD incidence and CVD mortality, focusing on the identification of subgroups expected to benefit most from initiating levothyroxine treatment for SCH, while minimizing risks of treatment.

## Author Contributions

LS wrote the first draft of the manuscript. All authors contributed to the article and approved the submitted version.

## Conflict of Interest

The authors declare that the research was conducted in the absence of any commercial or financial relationships that could be construed as a potential conflict of interest.
